# Embodied Conversational Agents Providing Motivational Interviewing to Improve Health-Related Behaviors: Scoping Review

**DOI:** 10.2196/52097

**Published:** 2023-12-08

**Authors:** José Mercado, Ismael Edrein Espinosa-Curiel, Juan Martínez-Miranda

**Affiliations:** 1 Unidad de Transferencia Tecnológica Tepic Centro de Investigación Científica y de Educación Superior de Ensenada Tepic, Nayarit Mexico

**Keywords:** embodied conversational agent, ECA, motivational interview, MI, health-related behaviors, virtual agents, mobile phone

## Abstract

**Background:**

Embodied conversational agents (ECAs) are advanced human-like interfaces that engage users in natural face-to-face conversations and interactions. These traits position ECAs as innovative tools for delivering interventions for promoting health-related behavior adoption. This includes motivational interviewing (MI), a therapeutic approach that combines brief interventions with motivational techniques to encourage the adoption of healthier behaviors.

**Objective:**

This study aims to identify the health issues addressed by ECAs delivering MI interventions, explore the key characteristics of these ECAs (eg, appearance, dialogue mechanism, emotional model), analyze the implementation of MI principles and techniques within ECAs, and examine the evaluation methods and primary outcomes of studies that use ECAs providing MI interventions.

**Methods:**

We conducted a scoping review following the PRISMA-ScR (Preferred Reporting Items for Systematic Reviews and Meta-Analyses Extension for Scoping Reviews) methodology. Our systematic search covered the PubMed, Scopus, IEEE Xplore, ACM Digital, and PsycINFO databases for papers published between January 2008 and December 2022. We included papers describing ECAs developed for delivering MI interventions targeting health-related behaviors and excluded articles that did not describe ECAs with human appearances and without the necessary evaluation or MI explanation. In a multistage process, 3 independent reviewers performed screening and data extraction, and the collected data were synthesized using a narrative approach.

**Results:**

The initial search identified 404 articles, of which 3.5% (n=14) were included in the review. ECAs primarily focused on reducing alcohol use (n=5, 36%), took on female representations (n=9, 64%), and gave limited consideration to user ethnicity (n=9, 64%). Most of them used rules-driven dialogue mechanisms (n=13, 93%), include emotional behavior to convey empathy (n=8, 57%) but without an automatic recognition of user emotions (n=12, 86%). Regarding MI implementation, of 14 studies, 3 (21%) covered all MI principles, 4 (29%) included all processes, and none covered all techniques. Most studies (8/14, 57%) conducted acceptability, usability, and user experience assessments, whereas a smaller proportion (4/14, 29%) used randomized controlled trials to evaluate behavior changes. Overall, the studies reported positive results regarding acceptability, usability, and user experience and showed promising outcomes in changes in attitudes, beliefs, motivation, and behavior.

**Conclusions:**

This study revealed significant advancements in the use of ECAs for delivering MI interventions aimed at promoting healthier behaviors over the past 15 years. However, this review emphasizes the need for a more in-depth exploration of ECA characteristics. In addition, there is a need for the enhanced integration of MI principles, processes, and techniques into ECAs. Although acceptability and usability have received considerable attention, there is a compelling argument for placing a stronger emphasis on assessing changes in attitudes, beliefs, motivation, and behavior. Consequently, inclusion of more randomized controlled trials is essential for comprehensive intervention evaluations.

## Introduction

### Background

Health-related behaviors refer to the actions and choices that individuals make that impact their physical, mental, and emotional well-being. These behaviors can either promote or compromise an individuals’ health [1]. Health risk behavior refers to an action performed by individuals that, because of its frequency or intensity, increases the likelihood of developing a disease or injury. It may occur whether or not the person is aware of the connection between the behavior and the associated health risks [2]. Conversely, positive health behaviors encompass actions that contribute to disease prevention, early detection of disease and disability, promotion and enhancement of overall health, and safeguarding against injury risk. These activities aim to maintain and improve one’s well-being [2].

Positive health behaviors are crucial for disease prevention, physical well-being, mental and emotional health, longevity and quality of life, productivity and performance, social interactions and relationships, and financial savings [2]. By proactively making positive behavioral changes, individuals can take control of their health, strive for long-term well-being, and prevent and reduce noncommunicable diseases (NCDs), widely acknowledged as chronic conditions, encompassing afflictions, such as cardiovascular disorders, neoplastic growths, persistent respiratory ailments, and diabetes [3]. This phenomenon arises because of the shared presence of behavioral risk factors among numerous NCDs, which comprise habits such as tobacco use, inadequate physical activity, unwholesome dietary patterns, and deleterious alcohol intake. NCDs account for 7 of the top 10 global causes of death [4,5] and cause 41 million deaths annually, equivalent to 74% of global deaths [3].

Promoting health prevention through improved health-related behaviors is a cost-effective and low-risk alternative to medication [6]. However, behavior changes can be challenging. Public health experts have developed a wide range of interventions to facilitate behavior change, including motivational interviewing (MI), which is recognized as highly effective [7]. MI combines brief interventions and motivational enhancement therapies [8] to encourage individuals to adopt healthier lifestyles [9,10]. MI aims to increase motivation and self-efficacy in adopting health-promoting behaviors using directive communication techniques and a person-centered approach. In MI, the counselors establish a collaborative, empathic, and nonjudgmental relationship with patients, using strategies such as reflective listening, strategic questioning, affirmations, and emphasizing patient autonomy to elicit *change talk* [11]. Change talk includes statements expressing an individual’s desires, abilities, reasons, needs, commitments, activations, and steps to modify behavior [11].

MI is guided by 4 *principles* that provide a framework for effective communication and behavior change in the counseling process [11-14]:

1. Express empathy: show genuine understanding and empathy toward the individual’s feelings and experiences, fostering a supportive and nonjudgmental environment.
2. Highlight discrepancies: help clients recognize discrepancies between their current behaviors and their desired goals, motivating them to consider change.
3. Roll with resistance: avoid confrontations and work with resistance by understanding its roots and navigating through it collaboratively.
4. Support self-efficacy: encourage belief in the client’s ability to make positive changes, empowering them to act toward their goals.

In addition to these principles, MI uses 4 interconnected *processes* that support individuals in exploring motivations, values, and abilities for positive behavior change [11-14]:

1. Engaging: develop a collaborative, trusting relationship with active listening and empathy to create a safe space for discussing changes.
2. Focusing: clarify behavior change goals by exploring motivations, values, and aspirations for intervention directions.
3. Evoking: elicit intrinsic motivation through reflective listening, affirmations, and open-ended questions to empower self-exploration.
4. Planning: collaboratively develop an achievable action plan with specific goals, strategies to overcome barriers, and tailored to individual strengths and preferences.

Finally, MI uses 4 essential *techniques* to facilitate effective communication and engagement [11-14]:

1. Open questions: encourage clients to express themselves freely and explore their thoughts and feelings in depth.
2. Affirmations: provide positive and supportive statements that acknowledge client strengths, efforts, and achievements, fostering trust and motivation.
3. Reflections: offer empathetic restatements of clients’ expressions, demonstrating active listening and understanding their perspectives.
4. Summaries: provide condensed recaps of the key points discussed, helping clients organize their thoughts and reinforcing important messages.

Accessibility of MI is limited by factors such as cost, logistics, social stigma, convenience, and counselor availability. Consequently, digital technology, including computers, smartphones, tablets, and the internet, is increasingly being used to deliver MI interventions and promote behavior change [15-17]. These technological approaches use various techniques such as chat rooms, automated responses, emoticons, decision balances, readiness rulers, and open-ended questions, which have demonstrated efficacy in modifying target behaviors [15]. Technology-based MI interventions have proven to be effective in promoting positive behavioral changes in chronic disease prevention and management. They offer advantages, such as reducing therapist burden and minimizing the need for extensive clinician training. Moreover, they improve access to care for underserved populations, address the stigma associated with disclosing risky behaviors [15], and provide cost-effective and easily accessible solutions for patients [16]. However, it is important to acknowledge that these interventions may have limitations in fulfilling critical components of MI [8], such as establishing rapport and demonstrating empathy [15,18].

To overcome these limitations, MI interventions delivered through embodied conversational agents (ECAs) have emerged as a promising and innovative approach. ECAs are advanced user interfaces that resemble humans and enable face-to-face conversations with users, incorporating verbal and nonverbal behaviors, such as body movements and facial expressions [19]. Interacting with ECAs can enhance user engagement and motivation, facilitating long-term use and maximizing their benefits [20]. ECAs can be developed for various platforms, including PCs and mobile devices, such as smartphones and tablets, enabling continuous use anytime, anywhere. The use of ECAs has been a subject of investigation in the field of clinical psychology, specifically in the context of various conditions, including autism spectrum disorders, major depressive disorder, anxiety disorders, posttraumatic stress disorder, psychotic disorders, and substance use disorders [21]. They have also been studied to support various health issues, such as overweight, obesity, diabetes, hypertension, and atrial fibrillation [22], and for coaching people in a healthy lifestyle, such as physical activity, nutrition, mindfulness, preconception care, stress, blood glucose monitoring, and sun protection [23]. The use and development of ECAs for health support have significantly increased because of advancements in personal devices (laptops, smartphones, or tablets) and computer techniques (3D game development, speech-to-text, text-to-speech, machine learning, and artificial intelligence) [22,24].

Using ECAs to deliver MI interventions in the context of improving health behaviors offers several advantages. ECAs can provide consistent, personalized, and nonjudgmental interventions that tailor support to individuals’ specific needs. They can adapt their communication style, tone, and visual cues to enhance engagement and motivation for behavior change [20]. Compared with text-based interfaces such as Technology-Delivered Adaptations of Motivational Interviewing (TAMI), ECAs have more personalized interactions and better relational skills, potentially providing stronger empathy and social connections [18]. Although TAMIs may have limited empathy conveyed through textual wording, ECAs can express empathy through verbal and nonverbal behaviors, resembling the approach of a human therapist [25]. Consequently, incorporating ECAs into MI interventions has the potential to yield improved outcomes [26-29].

Despite the potential of ECAs delivering MI interventions in improving health-related behaviors, there is limited understanding of their design, development, implementation, evaluation, and effectiveness. Previous reviews have explored ECAs in clinical psychology [21] and for coaching people in a healthy lifestyle [23]; however, they lack a specific analysis of ECA-delivered MI interventions. Similarly, although some reviews discuss TAMIs [15,16], they do not comprehensively examine ECA-delivered MI interventions. Therefore, a comprehensive review that specifically analyzes ECA-delivered MI interventions is needed.

### Objectives

The objective of this study was to conduct a scoping review with a focus on analyzing the use of ECAs to deliver MI. This study aimed to address the following questions:

1. Which health problems are addressed through ECAs providing MI?
2. What are the main characteristics of the ECAs used to provide MI interventions? (eg, device implementation, type of implementation, appearance, dialogue mechanism, and emotional model).
3. How are MI’s principles, processes, and techniques implemented in ECAs for MI interventions?
4. What evaluation protocols and measures are used and what are the primary reported results of interventions through ECAs providing MI?

By addressing these questions, this review enhances our understanding of the design, use, evaluation, and impact of ECAs delivering MI interventions, thereby contributing to the knowledge of digital interventions for improving health-related behaviors.

## Methods

### Overview

For our review, we adopted the PRISMA-ScR (Preferred Reporting Items for Systematic Reviews and Meta-Analyses extension for Scoping Reviews) methodology [30], a recognized framework for conducting systematic and unbiased reviews.

### Search Strategy

#### Search Sources

This review encompassed research papers published in the English language between January 2008 and December 2022, covering the last 15 years. The search encompassed prominent databases, including PubMed, Scopus (Elsevier), IEEE Xplore, ACM Digital, and PsycINFO. The selected databases collectively span the domains of medical, psychological, and computer science literature. An iterative approach was adopted to ensure a thorough search, whereby pertinent papers referenced within the retrieved articles were manually examined, further enhancing the comprehensiveness of the search process.

#### Search Terms

To develop the search query, the authors checked previous relevant reviews and made a list of words related to “embodied conversional agents” and “motivational interviewing.” We used a specific syntax for the query in each database, ensuring a consistent representation of the constructs: (1) ECAs (ie*,* virtual agent, digital health agent, and virtual assistant) and (2) MI (ie*,* brief MI, motivational interview, brief motivational intervention, and motivational intervention). The details of the exact terms used to search each database are presented in Multimedia Appendix 1.

### Study Eligibility Criteria

The inclusion criteria included papers describing ECAs developed for delivering MI interventions targeting health-related behaviors along with an evaluation procedure. In contrast, the exclusion criteria included papers that did not describe ECAs with a human appearance, did not use ECAs for improving health-related behaviors through MI, lacked evaluation, or lacked an explanation of MI implementation.

ECAs with a human appearance were given priority based on acceptability studies that indicate a preference for human agents over abstract, animal-like, and stylized (cartoon-like) agents [31-34]. In addition, it has been observed that the appearance of an ECA can influence the way users establish trust, communication, and engagement [19,25,35].

Furthermore, studies focusing solely on training health professionals in MI provision were excluded because the primary goal was to assess ECAs delivering MI for specific health-related problems. Papers without evaluations were also excluded, as they often represented ongoing work in the initial stages of ECA development. Similarly, papers lacking an explanation of MI implementation were not included, as accessing this information was necessary to address the implementation of MI in ECAs (question 3). We applied no restrictions on the study setting, study design, study outcome, month, and country of publication.

### Study Selection

We used a multistage screening process as follows. Initially, 3 reviewers (JM, IEE-C, and JM-M) conducted title and abstract screening. Articles that were included by all 3 reviewers proceeded to the second phase, in which the full text was thoroughly reviewed by the same 3 reviewers (JM, IEE-C, and JM-M) for final inclusion decisions. In instances in which multiple papers were associated with the same study or ECA, a hierarchical approach was implemented, favoring the most recent publication. This preference was maintained unless substantial disparities were evident in the assessment methodology, encompassing divergent metrics or distinct target demographic groups.

### Data Extraction

All studies that complied with the stipulated inclusion criteria were included in the review. The data extraction procedure was conducted by 3 independent reviewers (JM, IEE-C, and JM-M), and any discordance that arose was deliberated on through discourse to attain a unanimous consensus. Data extraction was centered on addressing the research objectives delineated in the Objectives subsection. A table template was developed to systematically extract and summarize relevant data such as ECA characteristics, MI implementation, evaluation, and principal outcomes. Multimedia Appendix 2 provides a description of the data extraction process. It is important to note that the data presented in this review were based solely on the information provided in each paper. If a specific piece of information was not mentioned or included in a paper, it was marked as “not mentioned.”

### Data Synthesis

Following data extraction from the included studies, a narrative approach was used to synthesize the data. The data synthesis was carried out by 3 independent reviewers (JM, IEE-C, and JM-M), and any discordances that arose were deliberated upon through discourse to attain a unanimous consensus. The synthesis of the data focused on summarizing and describing the health problems addressed, ECA characteristics (including appearance, dialogue mechanism, emotional model, deployment device, and implementation level), MI implementation (covering intervention type, principles, processes, and techniques), evaluation approach (including the protocol and measures used), and reported results. Microsoft Excel was used for the management of the synthesized data, whereas Mendeley was the tool of choice for reference management. The subsequent section provides a detailed description of the main findings of the included studies.

## Results

### Search Results

**Figure 1:**
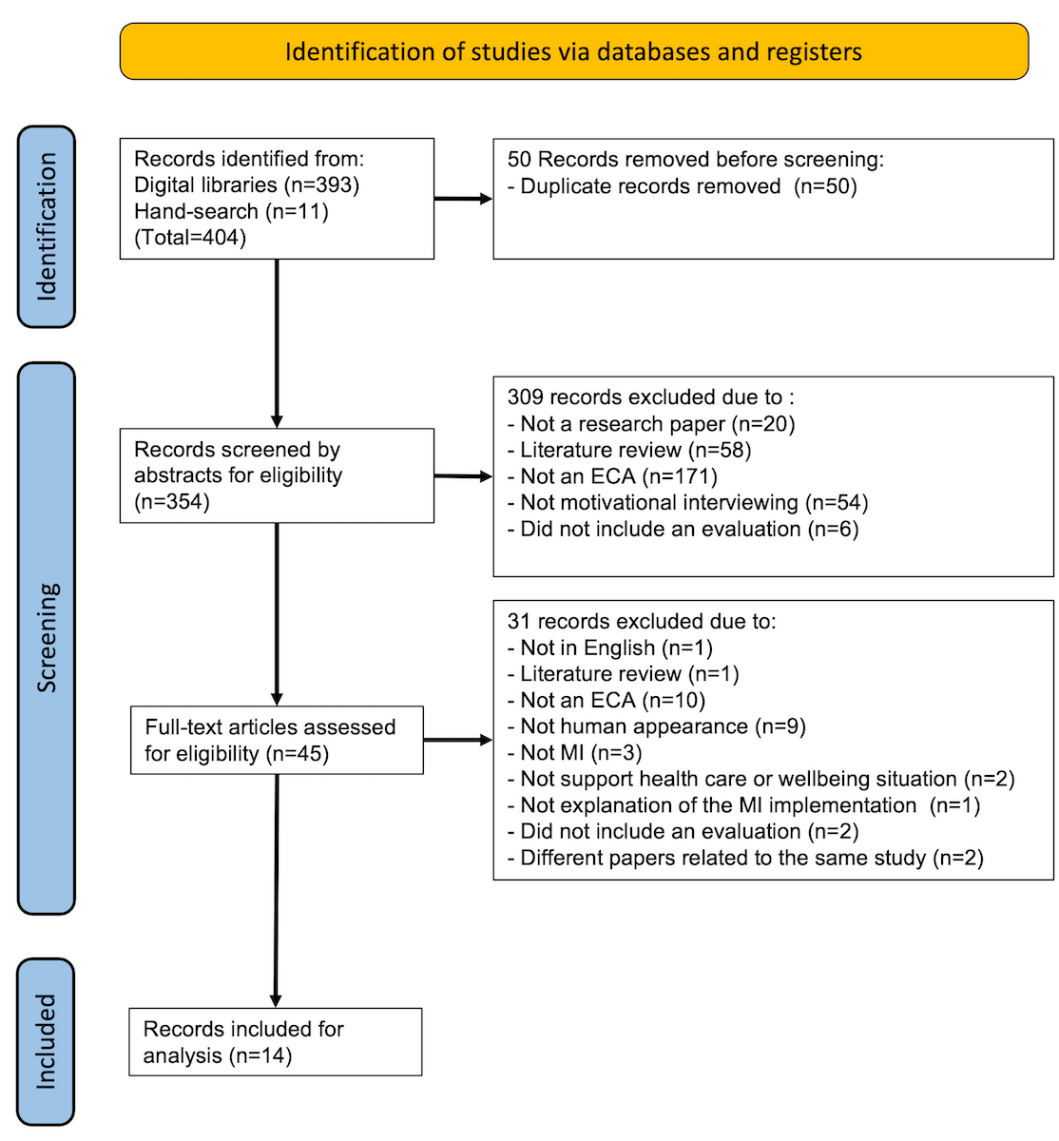
PRISMA-ScR (Preferred Reporting Items for Systematic Reviews and Meta-Analyses Extension for Scoping Reviews) flowchart of the study selection. ECA: embodied conversational agent; MI: motivational interviewing.

The search generated 404 entries, of which 97.3% (n=393) were retrieved from digital repositories and 2.7% (n=11) were manually acquired. The distribution across the individual libraries was as follows: 2.7% (11/404) from PubMed, 77.7% (314/404) from Scopus (Elsevier), 1% (4/404) from IEEE Xplore, 15.9% (64/404) from ACM Digital, and 0% (0/404) from PsycINFO. In the primary stage, 354 studies were assessed after eliminating duplicates. After scrutinizing the titles and abstracts, 87.3% (309/354) were excluded based on exclusion criteria. Subsequently, 45 articles underwent a full-text review, of which 31 (68.9%) were excluded. Thus, 14 studies were retained for further analysis. Figure 1 provides an overview of the review phase.

Regarding geographic distribution, most of the 14 studies originated from the United States (n=10, 71%), followed by the Netherlands (n=3, 21%) and Australia (n=1, 7%). The first study emerged in 2012, and the most recent in 2022. Notably, the number of studies has nearly doubled over the last 5 years. Table 1 provides a summarized overview of the general results, and Multimedia Appendix 3 provides details of the included studies [26-29,36-45]. The following sections detail the key findings that address the defined review objectives.

**Table 1:** Overview of the general results (n=14).

Characteristic			Publications, n (%)		References
**Publication type**					
	Journal article	9 (64)		[26-29,36-40]	
	Conference paper	5 (36)		[41-45]	
**Years**					
	2018-2022	9 (64)		[27,29,38-40,42-45]	
	2013-2017	4 (29)		[26,28,36,37]	
	2008-2012	1 (7)		[41]	
**Country of publication**					
	United States	10 (71)		[26,27,29,36,37,39,41-44]	
	The Netherlands	3 (21)		[28,38,40]	
	Australia	1 (7)		[45]	
**Health problem addressed**					
	Support reducing alcohol use	5 (36)		[29,36,37,39,41]	
	Promote physical activity and healthy eating	3 (21)		[28,42,43]	
	Promote women’s preconception health	2 (14)		[26,27]	
	Support cognitive learning	2 (14)		[38,40]	
	Counseling patients in medication-assisted treatment for opioid use disorder	1 (7)		[44]	
	Support brain injury rehabilitation decision-making processes	1 (7)		[45]	
**ECA^a^ appearance (gender)**					
	Female	9 (64)		[26,27,29,37,38,42-45]	
	Both (female and male)	5 (36)		[28,36,39-41]	
**ECA appearance (ethnicity)**					
	Did not match the ethnicity of the users	9 (64)		[28,29,37,38,40,42-45]	
	Match the ethnicity of the users	5 (36)		[26,27,36,39,41]	
**ECA dialogue mechanism**					
	Rules-driven	13 (93)		[26,27,29,36-45]	
	Predefined or sequential	1 (7)		[28]	
**ECA emotional model**					
	Only shows emotions	8 (57)		[27-29,37,39,42-44]	
	Recognize user’s emotion and show emotions	2 (14)		[36,41]	
	Not mentioned	2 (14)		[26,45]	
	Not implemented (a Wizard-of-Oz study)	2 (14)		[38,40]	
**ECA device implementation**					
	PC-based or web-based	10 (71)		[26-28,36,38-41,44,45]	
	Tablet or smartphone	4 (28)		[29,37,42,43]	
**ECA implementation level**					
	Full system	9 (64)		[26-29,36,37,39,42,43]	
	Prototype	3 (21)		[41,44,45]	
	Wizard-of-Oz	2 (14)		[38,40]	
**MI^b^ type**					
	MI	9 (64)		[26-28,38,40,42-45]	
	Brief MI	5 (36)		[29,36,37,39,41]	
**MI implementation**					
	As the core	11 (79)		[26-29,36,39,41-45]	
	As a component	3 (21)		[37,38,40]	
**MI principles implemented in the ECA**					
	Empathy	10 (71)		[26,27,36-43]	
	Discrepancy	10 (71)		[28,29,36-43]	
	Roll with the resistance	6 (43)		[36,39,41-43,45]	
	Self-efficacy	7 (50)		[28,38-40,42,43,45]	
**MI process implemented in the ECA**					
	Engaging	8 (57)		[26,27,36,38,40-43]	
	Focusing	13 (93)		[26-29,36-43,45]	
	Evoking	13 (93)		[26-29,36-43,45]	
	Planning	8 (57)		[26-28,36,39,41,44,45]	
	Tracking of behavior change	8 (57)		[26,27,36,41-45]	
**MI techniques implemented in the ECA**					
	Open questions	1 (7)		[28]	
	Affirmations	8 (57)		[29,36,38-43]	
	Reflections	10 (71)		[28,29,36,38-44]	
	Summaries	8 (57)		[28,29,36,37,41-43,45]	
	Provide information or advice	11 (79)		[26-29,37-40,42,43,45]	
**Evaluation protocol**					
	Pilot study	6 (43)		[36-40,44]	
	Randomized controlled trials	4 (29)		[26-29]	
	Preliminary user study	2 (14)		[41,45]	
	Quasi-experimental study	2 (14)		[42,43]	
**Evaluation measures**					
	Acceptability, usability, or user experience	13 (93)		[26-28,36-45]	
	Change on attitude, belief, or motivation	4 (29)		[38,40,42,43]	
	Change in behavior	4 (29)		[26-29]	
	Feasibility	3 (21)		[37,39,45]	
	Change on knowledge	2 (14)		[38,40]	

^a^ECA: embodied conversational agent.

^b^MI: motivational interviewing.

### Health Problems Addressed

In terms of the health problems addressed by ECAs, of the 14 studies, 5 (36%) focused on reducing unhealthy alcohol use [29,36,37,39,41]. In addition, 3 (21%) studies aimed to promote physical activity and healthy eating [28,42,43]. Moreover, 2 (14%) studies focused on promoting women’s preconception health [26,27], whereas another 2 (14%) studies focused on supporting cognitive learning [38,40]. Furthermore, 1 (7%) study centered on counseling patients in medication-assisted treatment for opioid use disorder [44], and 1 (7%) study aimed to support brain injury rehabilitation decision-making processes [45]. Among those focused on promoting physical activity and healthy eating, 2 of 3 specifically targeted promoting both physical activity and fruit and vegetable consumption [42,43], whereas the remaining 1 out of 3 solely focused on promoting physical activity [28].

The fact that the primary use of ECAs providing MI to support the reduction of alcohol use is not surprising, considering the strong support for MI’s efficacy (without ECAs) in addressing substance use, the very issue for which MI was originally designed [8]. Among the various drugs addressed, there is substantial evidence that traditional MI has a positive effect on reducing alcohol use [46]. This finding is consistent with previous reviews that reported TAMIs [15,16], wherein substance use behaviors, including alcohol use, were the most frequently addressed unhealthy change behaviors.

The next set of health-related behaviors most addressed by ECAs offering MI were those promoting physical activity, healthy eating, and women’s preconception health (5/14, 36%). Similar to the support for alcohol use reduction, this finding aligns with the MI literature, as the promotion of these healthy behaviors represents the second most addressed category of traditional MI [47]. In addition, previous systematic reviews of TAMIs [15,16] identified the promotion of these healthy behaviors as part of the second most commonly addressed health-related problems through these technological solutions.

The remaining reviewed studies addressed various other health-related problems, such as supporting cognitive learning, counseling patients in medication-assisted treatment for opioid use disorders, and providing support for brain injury rehabilitation for decision-making processes. The limited number of studies addressing these health problems could be attributed to their focus on more specific populations, necessitating further research to gather better evidence on the positive results of ECAs offering MI for such health-related issues. Notably, none of the reviewed studies explored the use of ECAs that provide MI to support the treatment or recovery of mental health problems. This represents a significant research opportunity, considering that MI is now increasingly used to address mental health disorders, such as anxiety, depression, and others [48]. Conversely, ECAs, when not providing MI, are also more commonly used for mental health treatments, including depression, anxiety, psychotic disorders, and other conditions [21]. Consequently, the development and evaluation of ECAs offering MI as a complement to psychotherapeutic interventions would be a relevant research endeavor to assess whether the outcomes for mental health problems can be improved compared with the current approach of using MI and ECAs separately.

### Characteristics of the ECAs

#### Overview

The main aim of ECAs is to implement natural and intuitive interactions with the users. These advanced interfaces are then equipped with a *body* that interacts multimodally by using *verbal*, para-verbal, and nonverbal behaviors. A socially intelligent manner of interaction with users should consider an adequate *emotional behavior* that helps to produce ECA’s reactions externalized by natural, expressive speech, and nonverbal behaviors [49]. On the basis of these ECA characteristics, we are interested in how the ECA’s appearance, the underlying dialogue mechanism, and the internal emotional model are designed and implemented in the included studies. Moreover, the degree of implementation (fully or not) and the type of devices in which the ECAs can be deployed were also analyzed. Table 2 presents the characteristics of ECAs.

**Table 2:** Characteristics of the embodied conversational agents.

Study	Appearance (gender)	Appearance (ethnicity)	Dialogue mechanism	Emotional model	Device implementation	Implementation level
Lisetti et al [41]	Both (female and male)	Matched the ethnicity of the users	Rules driven	Recognize user’s emotion and show emotions	PC	Prototype
Lisetti et al [36]	Both (female and male)	Matched the ethnicity of the users	Rules driven	Recognize user’s emotion and show emotions	PC	Full system
Friederichs et al [28]	Both (female and male)	Did not match the ethnicity of the users	Predefined or sequential	Only shows emotions	PC	Full system
Jack et al [26]	Female	Matched the ethnicity of the users	Rules driven	Not mentioned	PC	Full system
Schouten et al [38]	Female	Did not match the ethnicity of the users	Rules driven	Mentioned but not implemented (a Wizard-of-Oz study)	PC	Wizard-of-Oz
Olafsson et al [42]	Female	Did not match the ethnicity of the users	Rules driven	Only shows emotions	Tablet or smartphone	Full system
Tielman et al [37]	Female	Did not match the ethnicity of the users	Rules driven	Only shows emotions	Tablet or smartphone	Full system
Jack et al 2020 [27]	Female	Matched the ethnicity of the users	Rules driven	Only shows emotions	PC	Full system
Olafsson et al [43]	Female	Did not match the ethnicity of the users	Rules driven	Only shows emotions	Tablet or smartphone	Full system
Olafsson, et al [44]	Female	Did not match the ethnicity of the users	Rules driven	Only shows emotions	PC	Prototype
Boustani et al [39]	Both (female and male)	Matched the ethnicity of the users	Rules driven	Only shows emotions	PC	Full system
Hocking and Maeder [45]	Female	Did not match the ethnicity of the users	Rules driven	Not mentioned	PC	Prototype
Rubin et al [29]	Female	Did not match the ethnicity of the users	Rules driven	Only shows emotions	Tablet or smartphone	Full system
Schouten et al [40]	Both (female and male)	Did not match the ethnicity of the users	Rules driven	Mentioned but not implemented (a Wizard-of-Oz study)	PC	Wizard-of-Oz

#### ECA Appearance

The visual appearance of ECAs is a crucial aspect to be considered when they are used as the primary means of interaction. According to Cassell [19], the way an ECA looks captures a user’s attention and affects their cognitive and interactive abilities. ECAs have an advantage over chatbots because they can communicate nonverbally through facial expressions and body movements. Therefore, it is important to appropriately represent the embodiment to convey this type of communication. Moreover, simply having an embodied form in interactions with an agent enhances social outcomes, including motivation [50], the cornerstone of MI.

Of the 9 reviewed studies, only 4 (44%; 29% of the 14) mentioned a previous study as the basis for selecting the appearance of the ECA [29,37,38,40]. Conversely, none of the other studies (10/14, 71%) mentioned any previous study for selecting the ECA’s appearance [26-28,36,39,41-45]. In terms of the ECA’s gender, most ECAs use a female character (9/14, 64%) [26,27,29,37,38,42-45], whereas the rest of them present characters of both gender (5/14, 36%) [28,36,39-41], and no one provides only a male representation. Among the works that use a female representation, only 2 [37,38] reported a study about users’ preferences for ECA’s gender, where female ECAs were rated more positively than male ECAs. The greater use of female ECAs may be based on previous studies where young, female ECAs are preferred over male ECAs in health applications [51], on stereotypes where women are seen as more suitable for healing activities [35], or on previous studies using ECAs in psychotherapy where most users selected a female ECA over a male ECA when both options were offered [52]. Nevertheless, other studies point out the lack of consensus among users regarding the preferences of ECA’s gender in eHealth [25]. Thus, more studies are necessary to clearly identify the effect and influence of ECA’s gender on users, particularly when ECA is used to support behavior change.

Ethnicity is another demographic characteristic that influences the visual appearance of ECAs. Among the 14 reviewed studies, only 2 (14%) [26,27] developed the ECA’s appearance to match the ethnicity characteristics of the target users; 3 (21%) of them offer the option to select the preferred ECA’s ethnic background [36,39,41], whereas most of them did not consider the ethnicity of the target users (9/14, 64%) [28,29,37,38,40,42-45]. Although some previous studies highlighted the importance of ethnicity concordance between the ECA and the users in terms of preferences [53] and ECA’s task efficacy [54], others did not find evidence supporting ethnicity concordance as a predictor of perceived similarity (which, in turn, is associated with higher satisfaction, trust, and liking toward the ECA) [55] or an impact on higher social presence ratings [56]. Because of the lack of consensus regarding user-ECA ethnicity concordance, an effective strategy is to offer users a set of ECAs with different ethnic backgrounds, as provided in the studies by Lisetti et al [36], Boustani et al [39], and Lisetti [41]. This approach allows users the opportunity to choose the appearance with which they feel most comfortable, thereby increasing the adoption of this type of interface and, consequently, improving its efficacy toward behavior change.

#### ECA Dialogue Mechanism

Verbal communication is of the utmost importance in the successful implementation of MI techniques, encompassing activities such as posing open-ended questions, making affirmations, and summarizing key points. Therefore, it is crucial to evaluate how ECAs offering MI incorporate these techniques when interacting with users. Most ECAs in the reviewed studies (13/14, 93%) used a rules-driven dialogue mechanism and determined the next dialogue based on the progress of the session [26,27,29,36-45]. Only 1 of 14 (7%) studies used a predefined and sequential dialogue mechanism [28]. Furthermore, in 1 study that used a rule-driven dialogue mechanism, it also presented the first steps toward a machine learning–based dialogue engine and presented results on training a machine learning model to classify utterances [44].

Evidently, rule-driven approaches are widely used. In such approaches, the ECA’s responses to the user are determined based on predefined rules that consider information about the session’s progression. Alternatively, some studies opt for a more simplistic mechanism that relies on a prescripted dialogue for both ECA and user inputs. The prevalence of rule-driven approaches is not surprising, as natural language understanding, turn-taking management, and natural language generation present challenging obstacles. These challenges are prone to errors, which can have significant consequences in health applications, potentially leading to harmful outcomes [57].

Remarkably, only one of the reviewed studies introduced a dialogue mechanism that harnesses machine learning algorithms trained with annotated counseling sessions. This sophisticated mechanism automatically generates subsequent counseling actions at each turn of the dialogue, allowing unconstrained user speech [44]. The emergence of large language models**,** such as GPT-3/4**,** holds great promise in addressing many of the challenges in natural language processing and can significantly enhance MI sessions delivered by an ECA. Preliminary efforts have already been made to leverage GPT to automatically generate reflections on patients’ statements regarding their behavior [58]. Nevertheless, the use of large language models in psychotherapy necessitates careful consideration of certain factors, including risk detection, transparency, and bias, to ensure safe and effective therapeutic sessions [59].

#### ECA Emotional Model

In terms of ECAs’ behavior, the conveying of coherent emotional reactions toward user inputs is one of the characteristics of ECAs that facilitates the building of trust and a social bond with the user. There is evidence that artificial agents that express emotions and mood as part of their behavior are perceived to be more empathic, resulting in higher trust [60]. In addition, the emotions expressed by ECAs influence how users respond to them [61]. The expression of emotions has a communicative function: an emotion is used to communicate social feedback and empathy [62]. The communication of empathy in ECAs involves 2 processes: the recognition of the user’s emotion or emotions or experiences and the simulation of emotional cues in the ECA through, for example, facial expressions and body movements. It is widely accepted that empathy involves both cognitive and affective attributes [63]. Cognitive attributes of empathy involve understanding another individual’s experience and to communicating that understanding, whereas affective attributes involve emotional expressive responses to someone else’s display of emotions [64]. Thus, as empathy is one of the key principles of MI, it is important to know whether (and how) ECAs providing MI sessions implement emotional or empathic communication with the user.

Of the reviewed studies, 8 of 14 (57%) mentioned that the ECA included some mechanism to display empathy without explicitly recognizing the user’s emotions [27-29,37,39,42-44]. Another 2 of 14 (14%) participants recognized the user’s emotion and incorporated a mechanism to show empathy [36,41]. In 2 of 14 (14%) studies, it was not mentioned whether the ECA used any mechanism to display empathy [26,45]. Finally, 2 of 14 (14%) studies mentioned that the ECA displays empathic reactions but did not implement a mechanism to produce them, as they presented Wizard-of-Oz scenarios [38,40].

As can be seen, only one ECA demonstrates the capability to assess the user’s emotional state by analyzing real-time facial expressions. This ECA conveys empathy through techniques, such as reflective listening, head nodes, and facial expressions [36,41]. Other studies have implemented diverse emotional reactions and used empathic communication with the user using verbal utterances, such as simple reflections or emphasizing the positive aspects of the user’s current attitude [39,43]. In addition, nonverbal behaviors such as smiling, hand flips, and nodding were also used [39]. To provide effective empathic reactions, both cognitive and emotional, during interactions with the user, ECAs should incorporate a mechanism for automatically detecting and recognizing the user’s emotional state. The latest developments in multimodal interaction, where the automatic recognition of emotions is based on visual, audio, and speech recognition [65], and the nonverbal behavior of the ECA is generated through different channels [66], would benefit ECAs that provide MI sessions.

#### Device Deployment and Level of Implementation

The most commonly used devices to present ECAs were PCs (10/14, 71%) [26-28,36,38-41,44,45]. Of the 10 studies where the ECA was developed for PCs, 3 mentioned that the ECA was specifically developed for web access on PCs [28,39,45]. The remaining ECAs (4/14, 29%) were designed for use on mobile devices, such as tablets or smartphones [29,37,42,43]. It is evident that most of the reviewed ECAs were designed for use on PCs. Considering the significant advancements in smartphones and tablets, such as their high computational capabilities for rendering and animation tasks, it is expected that ECAs would be optimized for these devices to facilitate their anytime and anywhere use. However, a plausible explanation for this trend exists. The ECAs discussed in this review have primarily been developed for research purposes, where strict control over study participants is necessary. Thus, researchers could supply participants with the necessary devices and collect data under controlled conditions. This contrasts with commercial applications, in which mobile devices are deployed on a large scale. Nonetheless, future ECA developments should capitalize on smartphone capabilities, including integrated sensors for detecting user activities, as this can enhance and personalize MI-based recommendations. In terms of implementation level, of the 14 reviewed studies, 9 (64%) presented a full-system development [26-29,36,37,39,42,43]. In addition, 3 of 14 (21%) studies presented a prototype of the ECA [41,44,45], whereas the remaining 2 of 14 (14%) studies used a Wizard-of-Oz scenario [38,40]. In a Wizard-of-Oz scenario, a real person controls the movements and dialogues of the ECA. Prototypes and Wizard-of-Oz scenarios are frequently used during the development phase to assess the specific characteristics of the ECA. Given that some of the reviewed works describe preliminary and pilot studies, it is not surprising that the use of these prototypes serves as a foundation upon which more comprehensive versions can be built, informed by the findings derived from these initial evaluations.

### MI Implementation

#### MI Implementation and Theoretical Background

Among the 14 analyzed studies (Table 3), 9 (64%) used traditional MI [26-28,38,40,42-45], whereas 5 (36%) used brief MIs [29,36,37,39,41]. Within the subset of brief MI studies, the Drinker’s Check-Up method was used in 3 of 5 studies [36,39,41], the Screening, Brief Intervention, and Referral to Treatment model in 1 of 5 studies [29], and the Feedback, Responsibility, Advice, Menu, Empathy, and Self-Efficacy model in 1 of 5 studies [37]. MI was implemented as the core of ECAs in 11 of 14 (79%) studies [26-29,36,39,41-45] and as a component in 3 of 14 (21%) studies [37,38,40].

The transtheoretical model was explicitly specified in 8 of 14 (57%) studies to understand the stages of change [26,27,29,36,37,41-43]. In addition, other theories were incorporated in 7 of 14 (50%) studies [26,38-40,42,44,45] to support specific intervention activities, including Self-Determination Theory [45], Formal Scaffolding Theory [38,40], Small Talk Theory [38,40], and Provider-Patient Communication Theory [26]. One (7%) study used techniques such as emotional recognition and mindfulness to enhance intervention effectiveness [44].

Overall, most of the reviewed studies used traditional MI, whereas a smaller proportion used brief MI. The choice between these 2 approaches might be influenced by various factors, such as setting, time constraints, available resources, and individual or population needs. Traditional MI allows for comprehensive exploration, whereas brief MI uses a more condensed and targeted approach. MI was identified as the central component in 11 (79%) studies, often combined with other elements in 3 (21%) studies. Half of the studies (n=7, 50%) incorporated supplementary theories to support their intervention. Further research is required to evaluate the effectiveness of ECAs using traditional MI and brief MI in diverse settings and populations. In addition, exploring the integration of MI with other techniques or theories to enhance its effectiveness and address individual needs requires further research and development.

**Table 3:** Motivational interviewing (MI) implementation characteristics.

Study	MI type	MI implementation level	MI principles	MI processes	MI techniques
Lisetti et al [41]	Brief MI	Complete	Empathy, develop discrepancy, and roll with resistance	Engaging, focusing, evoking, planning, and tracking of behavior change	Affirmations, reflective listening, and summaries
Lisetti et al [36]	Brief MI	Complete	Empathy, develop discrepancy, and roll with resistance	Engaging, focusing, evoking, planning, and tracking of behavior change	Affirmations, reflective listening, and summaries
Friederichs et al [28]	MI	Complete	Develop discrepancy and support self-efficacy	Focusing, evoking, and planning	Open questions, reflective listening, summaries, and provide information or advice
Jack et al [26]	MI	Complete	Express empathy	Engaging, focusing, evoking, planning, and tracking of behavior change	Provide information or advice
Schouten et al [38]	MI	Component	Empathy, develop discrepancy and self-efficacy	Engaging, focusing, and evoking	Affirmations, reflective listening, and provide information or advice
Olafssonet al [42]	MI	Complete	Empathy, develop discrepancy, roll with resistance, and self-efficacy	Engaging, focusing, evoking, and tracking of behavior change	Affirmations, reflective listening, summaries, and provide information or advice
Tielmanet al [37]	Brief MI	Component	Express empathy and develop discrepancy	Focusing and evoking	Summaries and provide information or advice
Jack et al [27]	MI	Complete	Express empathy	Engaging, focusing, evoking, planning, and tracking of behavior change	Provide information or advice
Olafsson et al [43]	MI	Complete	Empathy, develop discrepancy, roll with resistance, and self-efficacy	Engaging, focusing, evoking, and tracking of behavior change	Affirmations, reflective listening, summaries, and provide information or advice
Olafsson et al [44]	MI	Complete	Not specified	Planning and tracking of behavior change	Reflective listening
Boustani et al [39]	Brief MI	Complete	Empathy, develop discrepancy, roll with resistance, and self-efficacy	Focusing, evoking, and planning	Affirmations, reflective listening, and provide information or advice
Hocking and Maeder [45]	MI	Complete	Roll with resistance and self-efficacy	Focusing, evoking, planning, and tracking of behavior change	Summaries and provide information or advice
Rubin et al [29]	Brief MI	Complete	Develop discrepancy	Focusing and evoking	Affirmations, reflective listening, summaries, and provide information or advice
Schouten et al [40]	MI	Component	Empathy, develop discrepancy, and self-efficacy	Engaging, focusing, and evoking	Affirmations, reflective listening, and provide information or advice

#### MI Core Principles, Processes, and Techniques Implemented in the ECA

When analyzing the core principles of MI (empathy, discrepancy, rolling with resistance, and self-efficacy) [11-14] in the 14 reviewed studies, the following frequencies were observed (Table 3): 3 of 14 (21%) studies included all 4 principles [39,42,43], 4 of 14 (29%) studies included 3 principles [36,38,40,41], 3 of 14 (21%) studies included 2 principles [28,37,45], and 4 of 14 (29%) studies included 1 or no principles [26,27,29,44]. The specific frequencies for each principle were as follows: empathy was expressed in 10 of 14 (71%) studies [26,27,36-43], discrepancy was developed in 10 of 14 (71%) studies [28,29,36-43], rolling with resistance was addressed in 6 of 14 (43%) studies [36,39,41-43,45], and self-efficacy was supported in 7 of 14 (50%) studies [28,38-40,42,43,45]. These results indicate significant variation in the inclusion of core MI principles. Notably, only 21% of the studies encompassed all 4 principles, and 50% included 2 or fewer principles. These findings highlight a deficiency in adhering to MI principles, which are crucial for maintaining fidelity and integrity of the interventions, as they form the foundation for behavior change and motivation. Self-efficacy and the principle of rolling with resistance exhibited the lowest inclusion rate. This suggests a failure to address resistance or ambivalence toward change, emphasizing the need for activities that can enhance beliefs in positive behavior changes.

Regarding the basic processes of MI [11-14] (engaging, focusing, evoking, and planning), the frequencies observed in the 14 studies were as follows (Table 3): 4 of 14 (29%) studies included all 4 processes [26,27,36,41], 7 of 14 (50%) studies included 3 processes [28,38-40,42,43,45], 2 of 14 (14%) studies included 2 processes [29,37], and 1 of 14 (7%) studies included 1 process [44]. The specific frequencies for each process were as follows: the engaging process was included in 8 of 14 (57%) studies [26,27,36,38,40-43], the focusing process was present in 13 of 14 (93%) studies [26-29,36-43,45], the evoking process was incorporated in 13 of 14 (93%) studies [26-29,36-43,45], and the planning process was included in 8 of 14 (57%) studies [26-28,36,39,41,44,45]. In addition, the ECAs introduced a supplementary process, namely, tracking of behavior change, which was found in 8 of 14 (57%) studies [26,27,36,41-45]. Similarly, the implementation of MI processes in ECAs showed a varied inclusion. Only 29% of the studies encompassed all 4 processes, with 21% including 2 or fewer. The omission of any process results in an incomplete intervention that lacks the necessary components for effective MI. Surprisingly, there is a noticeable lack of engagement and planning processes in many cases. The absence of an engaging process hinders collaborative relationships and motivation for change, whereas the lack of planning activities limits goal setting and the identification of effective strategies.

Regarding the basic techniques of MI [11-14] (open questions, affirmations, reflections, and summaries), the frequencies observed in the 14 studies were as follows (Table 3): none included all 4 techniques [36,41], 6 of 14 (43%) studies included 3 techniques [28,29,36,41-43], 3 of 14 (21%) studies included 2 techniques [38-40], and 5 of 14 (36%) studies included one or no technique [26,27,37,44,45]. The specific frequencies for each technique were as follows: open questions were used in 1 of 14 (7%) studies [28], affirmations were present in 8 of 14 (57%) studies [29,36,38-43], reflections were used in 10 of 14 (71%) [28,29,36,38-44] studies, and summaries were included in 8 of 14 (57%) studies [28,29,36,37,41-43,45]. In addition, the ECAs introduced a supplementary technique, namely, information providing and advising, which were found in 11 of 14 (79%) studies [26-29,37-40,42,43,45].

Similarly, there was variation in which MI techniques were included in the ECAs. Notably, none of the studies included all 4 techniques, with 57% (8/14) including 2 or fewer techniques. The findings highlight a lack of adherence to MI techniques, which can impact engagement, effectiveness, and an individual’s active participation, autonomy, and self-reflection. The absence of open-ended questions limits the exploration of perspectives, thoughts, and feelings. Missing affirmations hinder the recognition of strengths, efforts, and positive attributes. The absence of summaries inhibits key point consolidation and understanding of thoughts, feelings, and motivations. One of the adaptations included in most of the ECAs is information providing and advising in 79% (11/14) of the studies.

In general, most articles focused on specific features of MI rather than encompassing the entire approach. Furthermore, most studies recognized the use of MI principles, processes, and techniques but lacked explicit details on how these elements were specifically integrated into ECAs. This finding aligns with the review by Shingleton and Palfai [15]. The absence of certain MI principles, processes, and techniques in ECAs can be attributed to the complexity of MI and the technological constraints. MI demands expertise and skill, making its translation into ECA challenging. Capturing the nuanced aspects of MI within technological limitations is demanding. Technological constraints in natural language processing, context understanding, real-time adaptability, and supporting behavior change complexity hinder the development of ECAs that fully embrace MI. For example, an ECA’s natural language processing may struggle with open-ended questions or reflections. Fixed programming and limited adaptability hinder client-centered MI interventions. The integration of advancements in natural language processing, context understanding, machine learning, and artificial intelligence is necessary to overcome these constraints. Further research and development are essential to enhance the capabilities of ECAs and provide authentic support for MI-based behavior changes.

### Evaluation Procedures and Main Results Reported

The studies assessed ECAs using diverse methods (see details in Table 4 and in Multimedia Appendix 4), including preliminary user studies (2/14, 14%) [41,45], pilot studies (6/14, 43%) [36-40,44], quasi-experimental studies (2/14, 14%) [42,43], and randomized controlled trials (RCT) (4/14, 29%) [26-29]. Among all the studies, 3 of 14 (21%) used feasibility measures as the evaluation criteria [37,39,45]. In addition, 13 of 14 (93%) used measures to assess the acceptability, usability, or user experience [26-28,36-45]. Furthermore, 2 of 14 (14%) studies used measures to evaluate changes in knowledge [38,40]; 4 of 14 (29%) measured changes in attitude, belief, or motivation [38,40,42,43]; and 4 of 14 (29%) evaluated changes in behavior [26-29].

Preliminary user studies have reported positive results concerning feasibility, acceptability, usability, and user experience evaluations. For instance, Hocking and Maeder [45] reported positive therapeutic relevance and probable usability in the initial tests of the ECA prototype RehabChat. They also validated the ECA’s conversation and discourse structuring as appropriate. In addition, the results reported by Lisetti et al [41] indicated that the use of ECAs for health-promoting interventions might lead to higher user engagement, lower attrition rates, and overall increased divulgation of sensitive issues because of improved confidentiality.

In addition, the results of the pilot studies indicated that ECAs were developed and evaluated with a set of participants with respect to feasibility, acceptability, user experience, and usability. Of these studies, 2 [38,40] also evaluated changes in knowledge and changes in attitudes, beliefs, and motivations. Overall, users have a positive reaction and acceptance of ECAs, and the results also indicate that ECAs could have a significant impact on users’ motivation to continue using them for interventions.

**Table 4:** Evaluation protocols and measures.

Study	Level of the evaluation protocol	Participants, n	Participants profile	Evaluation measures
Lisetti et al [41]	Preliminary user study	11	Individuals with different levels of technicality	Acceptability, usability, or user experience
Lisetti et al [36]	Pilot study	81	Volunteer university students	Acceptability, usability, or user experience
Friederichs et al [28]	RCT^a^ study	958	Dutch adults (aged 18-70 years)	Acceptability, usability, or user experience Change in behavior
Jack et al [26]	RCT study	100	Women who self-identified as African American or Black, were aged 18-34 years, and self-reported as not currently pregnant	Acceptability, usability, or user experience Change in behavior
Schouten et al [38]	Pilot study	34	Participants from 5 reading and writing classes throughout the Netherlands, with ages ranging from 19 to 64 years (mean 41.3, SD 115.1)	Acceptability, usability, or user experience Change in knowledge Change in attitude, belief, or motivation
Olafsson et al [42]	Quasi-experimental study	39	Individuals aged at least 21 years, able to speak and read English, in 1 of the 3 first stages of change	Acceptability, usability, or user experience Change in attitude, belief, or motivation
Tielman et al [37]	Pilot study	Pilot 1: 20, pilot 2: 30	Participants are all males, aged 27-82 years	Feasibility Acceptability, usability, or user experience
Jack et al [27]	RCT study	528	Self-reported to be African American or Black or both, aged 18-34 years, and currently not pregnant	Acceptability, usability, or user experience Change in behavior
Olafsson et al [43]	Quasi-experimental study	15	Individuals aged at least ≥21 years, able to speak and read English, and self-report as being in one of the first 3 stages of change	Acceptability, usability, or user experience Change in attitude, belief, or motivation
Olafsson et al [44]	Pilot study	23	Participants were recruited at an addiction treatment hospital in Reykjavik (Iceland) and a treatment facility in Boston (Massachusetts), with an eligibility criterion of being in medication-assisted treatment for opioid use. Individuals aged ranging from 23 to 67 years	Acceptability, usability, or user experience
Boustani et al [39]	Pilot study	51	Participants were aged 21-55 years, had to have engaged in heavy drinking (consumed 5 drinks in one sitting at least once in the past year), not currently be receiving treatment for their AUD^b^, and not have a medical condition for which alcohol use would be contraindicated	Feasibility Acceptability, usability, or user experience
Hocking and Maeder [45]	Preliminary user study	Alpha testing: 3, beta testing: 11	Alpha testing: 3 senior academic researchers; beta testing: Flinders University PhD candidates and academic staff, with experience in digital health technology or health care	Feasibility Acceptability, usability, or user experience
Rubin et al [29]	RCT study	178	Veterans, with a positive AUDIT-C^c^ screening, drinking within the past 30 days. Participants were divided on 2 groups:—group 1, age: 49.52 years (SD 17.34), 79 male, 10 female;—group 2, age: 53.90 (SD 18.39), 79 male, 10 female	Change in behavior
Schouten et al [40]	Pilot study	12	Participants were recruited in several language classes throughout the Netherlands; 6 men and 6 women participated, with ages ranging from 30 to 63 (mean 48.2, SD 10.5)	Acceptability, usability, or user experience Change in knowledge Change in attitude, belief, or motivation

**^a^**RCT: randomized controlled trial.

^b^AUD: alcohol use disorder.

^c^AUDIT-C: Alcohol Use Disorder Identification Test-Concise.

For example, Boustani et al’s [39] study revealed participants’ highly positive experiences with ECA, including engagement, acceptance, utility perception, and intent to reuse. Similarly, in the study presented by Olafsson et al [44], participants had a mostly positive response to the ECA, reporting a high degree of confidence in the agent and willingness to work with her another time, and they were mostly satisfied with the overall interaction.

The results from the study by Tielman et al [37] showed that participants reported being comfortable with the ECA and were willing to give ECA sensitive information on alcohol use. As a result, the ECA was considered an effective medium for substance use screening in primary care. Lisetti et al [36] reported users’ overall acceptance of their ECA (ODVIC) about the effect of the character’s empathic interaction, indicating that the use of the ECA could have an important impact on users’ motivation to continue using computerized behavior change interventions, leading to the adoption and maintenance of healthy lifestyles in the long term.

In a study by Schouten et al [38], learners effectively interacted with the coach and completed the exercises. Although no significant differences emerged between the prototypes, improvements were recommended for affective and social support. In a subsequent study [40], improved ECA (VESSEL) showed beneficial effects for low-literacy learners, enhancing positive affect, user engagement, and self-efficacy.

The 2 papers reporting quasi-experimental studies show the positive outcomes of using ECAs for MI, primarily in terms of changes in attitude, belief, or motivation. These studies also reported positive results regarding acceptability, usability, and user experience. For instance, Olafsson et al [42] reported that both interventions were effective at changing participants’ motivation, confidence, and self-efficacy to improve physical activity and fruit and vegetable consumption behaviors. Furthermore, the participants expressed high levels of satisfaction with the agents and interventions. In addition, the results from Olafsson et al [43] indicated that using an ECA that uses affiliative and topically appropriate humor in MI could enhance people’s motivation to adopt a healthier lifestyle about eating and exercising. The presence of humor during interactions with the ECA encourages engagement with it.

Finally, the reported results of RCT studies were generally positive, with most reporting substantial differences in behavioral change among the intervention with the ECA compared with the control groups. Some of these studies have also reported positive results regarding acceptability, usability, and user experience. For instance, Jack et al [26] reported that interacting with Gabby (their ECA) was highly related with a higher proportion and an overall greater percentage of women’s preconception health risks being resolved after 6 months compared with women who were not in touch with Gabby. In a later and more extended study, when comparing Gabby to the control group, Jack et al [27] confirmed a rise in the reported number of preconception care risks, achieving the action or maintenance stage of the transtheoretical model. The study also confirmed that these behavioral changes were maintained at 12 months.

Rubin et al [29] reported that their ECA offered MI satisfactorily and sent more patients to specialty treatment. They discovered that the ECA may intervene with patients who have less severe drinking problems, without adding to the primary care burden. The authors also stated that using ECAs for MI could result in a more standardized use of psychometric tests and evaluation procedures. However, only one RCT [28] reported no differences in physical activity levels between those receiving ECA and text-based interventions. Nevertheless, compared with the control condition, both therapies used in the study significantly improved self-identified physical activity after 1 month. The authors mentioned that the intervention was effective in promoting behavioral changes. The lack of significant distinctions from the control group could be attributed to their ECA’s incapacity to react with gestures to the user’s condition and input. They proposed addressing this issue in future work to enhance the effectiveness of ECA intervention.

Overall, all reviewed studies reported positive results. Most of them conducted evaluations of ECAs through preliminary or pilot studies (8/14, 57%) and focused on assessing feasibility, acceptability, usability, or user experience. These findings provide evidence that ECAs providing MI generally receive positive reactions and acceptance from their users. Moreover, the results indicated that ECAs could have a significant impact on users’ motivation to continue using them for interventions, potentially supporting long-term use. Another important result is that some studies addressed the initial step of screening for substance use by the ECA [37,41,44] and noted that ECAs could effectively serve as a medium for screening because of the increased confidentiality of participants with the virtual agent. Participants reported feeling comfortable and expressed high levels of trust in these ECAs, which facilitated disclosure of sensitive issues. However, further studies are needed to evaluate their efficacy in specific target populations. Such evaluations are fundamental to demonstrating the impact of ECAs on people’s health and well-being, aligning with the World Health Organization global strategy on digital health [67], and ensuring effective adoption and appropriation of ECAs providing MI as digital health technologies.

The 2 quasi-experimental studies showed positive and promising outcomes concerning changes in attitudes, beliefs, or motivation to promote physical activity and healthy eating. However, as participants in these studies only underwent the intervention during 1 session, it is necessary to assess the effects of a long-term intervention to gain a more comprehensive understanding of these results and to determine whether ECA can also lead to improvements in behavioral changes. Finally, the reported RCT studies (4/14, 29%) demonstrated positive outcomes in terms of behavioral changes, promoting physical activity and healthy eating, enhancing women’s preconception health, and supporting a reduction in alcohol use. These results show promising evidence of the efficacy of ECAs; however, conducting more RCTs would provide a better understanding of the ECAs’ impact of ECAs on behavioral changes when providing MI. In addition, these studies should include follow-up evaluations to assess whether behavioral changes are sustained in the long term.

## Discussion

### Principal Findings

This study explored the development and use of ECAs to deliver MI to promote health-related behaviors. Following the PRISMA-ScR methodology, we identified a total of 404 articles, of which 14 met the inclusion criteria for this scoping review. Among these, 5 were published between 2012 and 2018, whereas 9 were published between 2019 and 2022. It is noteworthy that all studies included in this review originated from 3 countries: the United States, the Netherlands, and Australia. These studies addressed distinct health-related issues, mainly the reduction of alcohol use and the promotion of physical activity and healthy eating.

Different characteristics of the development of ECAs have been identified in this study. Concerning the representation of ECAs’ gender, most studies used female characters. Regarding ethnicity, 2 studies tailored the ECA’s appearance to match the ethnicity characteristics of the target users, 9 studies did not consider the ethnic background of the users, and 3 studies offered users the option of selecting their preferred ECA’s ethnic background. The vast majority of the ECAs implemented rules-driven dialogue mechanisms, although one of them presented the initial steps toward a machine learning–based dialogue engine. Moreover, 10 studies developed ECAs with mechanisms to display empathy, although they did not explicitly recognize the user’s emotions. Only 2 studies mentioned that their ECAs could recognize the user’s emotions and used this information to display empathy. In terms of the implementation level, 9 studies described a full system, 3 studies presented a prototype of the ECA, and 2 studies used a Wizard-of-Oz scenario in which a real person controlled the dialogue and behavior of the ECA. Finally, most ECAs were designed to be deployed on a PC, whereas 4 were specifically developed for use on mobile devices.

When assessing how MI is implemented by ECAs, 9 (64%) studies used traditional MI, whereas 5 (36%) studies used brief MI. Regarding the specific implementation details of the MI principles, processes, and techniques, only 3 (21%) of the included studies incorporated all 4 MI. A total of 4 (29%) studies encompassed all 4 MI processes, but none included all 4 MI techniques. Instead, 12 (86%) studies integrated 1 to 3 MI techniques, whereas 2 (14%) studies did not incorporate any MI techniques. Regarding the evaluation methods used for ECAs, 2 studies conducted preliminary user studies, 6 described pilot studies, 2 used quasi-experimental studies, and 4 conducted RCTs. Overall, the findings of these diverse studies are favorable, supporting the use of ECAs to deliver MI for various health-related issues. However, it is important to note that additional follow-up evaluations are necessary to determine whether the levels of acceptability and observed changes in attitudes, beliefs, or motivation to change persist over the long term.

### Comparison With Previous Studies

Among the works reviewed, previous reviews have explored ECAs in different domains, including clinical psychology [21] and ECAs for coaching people in a healthy lifestyle [23]. Notably, these reviews have concentrated on specific problem domains. Provoost et al [21] exclusively examined studies related to clinical psychology with a well-defined focus on conditions, such as autism, depression, anxiety, posttraumatic stress disorder, psychosis, and substance use. Kramer et al [23] focused on the group of lifestyle behaviors, identifying areas such as physical activity, nutrition, mindfulness, preconception care, stress, blood glucose monitoring, and sun protection as targets for ECAs. Both reviews primarily emphasized the characteristics of ECAs and the outcomes derived from evaluating these ECAs. It is worth noting that although Provoost et al [21] provided some insight into the specific interventions used for each condition, Kramer et al [23] only briefly mentioned the types of interventions used in the studies without conducting a detailed analysis of their characteristics or functions. Consequently, there is a conspicuous gap in both reviews as neither offers a comprehensive analysis of ECA-delivered MI interventions. Incorporating a dual analysis, one study focusing on the ECA characteristics and another study focusing on the interventions used would be beneficial for a diverse group of researchers seeking a deeper understanding of the current developments and applications of ECAs for delivering MI interventions.

In contrast, previous reviews have discussed TAMIs [15,16]. Their principal objective was to explore the landscape of technologies used for MI and assess their methods and effectiveness. Interestingly, both reviews identified only a limited number of studies using avatars for MI, yet the analyses of these studies lacked specific details regarding the components of ECAs. The absence of studies using ECAs may be attributed to the fact that both reviews primarily sourced their studies from medical and psychological databases such as PubMed, PsycINFO, and CINAHL, potentially missing relevant works that could be found in computer science and technological sources.

In this scoping review, we narrow our focus exclusively to ECAs, aiming to gain a better understanding of this technology, which possesses numerous features capable of enhancing and emulating traditional MI, as mentioned earlier. Moreover, we broadened our search horizons, encompassing not only medical and psychological databases but also delving into technology and computer science databases. Consequently, the primary objective of this study was to conduct a scoping review of ECAs implementing MI, with the intent of providing details of the attributes and characteristics unique to this category of ECAs.

### Strengths and Limitations

#### Strengths

To the best of our knowledge, this is the first review based on PRISMA-ScR guidelines with a specific focus on the use of ECAs for delivering MI to enhance health-related behaviors. This pioneering study offers a valuable resource for researchers, computer scientists, and health care leaders interested in leveraging ECA technology to deliver MI to promote positive health-related behaviors. It is worth noting that our review maintains inclusivity, as we imposed no constraints on study design, country of publication, or study settings. We also conducted a backward-referencing check, which led to the identification of additional studies. To mitigate selection bias, we implemented a rigorous process in which 3 independent reviewers performed study selection and data extraction. Significantly, consensus was achieved among the reviewers at all stages, and complete agreement was reached following thorough discussions. This rigorous approach enhanced the reliability and validity of our findings, thereby increasing confidence in the outcomes of this review.

#### Limitations

Our study has several limitations. First, our search was restricted to articles published in the English language, limited to peer-reviewed databases, and we did not extensively search for gray literature, which may have restricted the comprehensiveness of our findings. Second, our database selection favored those recognized for their comprehensive coverage of the medical, psychological, and computer science literature, which led to the exclusion of other widely used databases. This exclusion was primarily due to their thematic specialization (eg, education) or substantial overlap with our chosen databases. However, it is important to note that the expected impact of this exclusion on the results is minimal, and we mitigated this by searching the reference lists of all included studies. Third, our inclusion criteria specifically targeted studies that included user evaluations, potentially resulting in the omission of ongoing research. Fourth, because of the scope of this study, we did not conduct a quality assessment of the included papers. Fifth, in certain studies, the descriptions of ECAs and explanations of MI implementation were insufficiently detailed. This lack of clarity in ECA descriptions and MI implementation could pose challenges for researchers attempting to extract ECA components and to understand MI principles, processes, and techniques. In addition, several studies have integrated supplementary techniques, but it is unclear how they were integrated with MI or their impact on the intervention. Finally, the studies exhibited significant heterogeneity in design, objectives, and data collection methods. This diversity challenges our ability to compare effectiveness, summarize findings, and draw clear conclusions from data. Despite these limitations, this review highlights key gaps in the literature and provides a solid foundation for future research optimization.

### Future Directions

This study focuses on a scoping review of ECAs offering MI to support the adoption of health-related behaviors. Although most reported evaluations yield positive results, several opportunities remain for enhancing the design and implementation of ECAs with the capability to provide MI. Further studies are essential to gain a more profound understanding of how appearance of an ECA influences user adoption and acceptability. Given the ongoing lack of consensus regarding the effects of ECA gender and ethnicity, it is advisable to provide users with various ECA options or the ability to customize these characteristics, enhancing their suitability for diverse user groups. Considering the core role of empathy in MI, future development of ECAs should prioritize the implementation of multimodal interfaces, allowing for the automatic interpretation of user emotions and the generation of appropriate emotional responses within the ECA. In terms of MI implementation, future studies should explicitly report which principles, processes, and techniques are incorporated and how they are delivered by ECA. This level of detail will facilitate more meaningful comparisons across different studies and provide a clearer understanding of the differences in the results. Finally, additional RCTs that include follow-up evaluations are imperative to determine whether positive changes in attitudes and behaviors are sustained in the long term.

### Conclusions

This scoping review highlights significant progress in the use of ECAs for health care–focused MI interventions over the past 15 years. These interventions aim to encourage healthier behaviors, such as reducing alcohol use, increasing physical activity, improving dietary habits, and enhancing women’s preconception health. However, several challenges remain in this field. One notable trend is the prevalence of ECAs with a female appearance, often overlooking user characteristics and lacking automatic emotion recognition, and ECA’s dialogues heavily rely on rule-based systems. Furthermore, the reviewed studies often provided inadequate details regarding the integration of MI principles, processes, and techniques into ECAs. Although there has been substantial focus on assessing feasibility, acceptability, usability, and user experience, less emphasis has been placed on understanding changes in attitudes, beliefs, motivation, and behavior. Future research should explore how the characteristics of ECAs influence user perceptions and outcomes to fully understand the potential of ECAs in delivering MI for health-related behaviors. In addition, studies should offer comprehensive insights into the integration of MI principles. Moreover, it is crucial to conduct additional RCTs to thoroughly assess the effectiveness of these interventions.

## Appendix

Multimedia Appendix 1 Keywords used to find studies.

Multimedia Appendix 2 Description of the data extraction fields.

Multimedia Appendix 3 Details of the included studies.

Multimedia Appendix 4 Extended details of the evaluation protocol and main reported results.

Multimedia Appendix 5 PRISMA-ScR checklist.

## Abbreviations

ECA: embodied conversational agent
MI: motivational interviewing
NCD: noncommunicable disease
TAMI: Technology-Delivered Adaptations of Motivational Interviewing

## Data Availability

The data sets generated during and/or analyzed during this study are included in this published article or are available from the corresponding author on reasonable request.
