# Supplementary material for: Embodied Conversational Agents Providing Motivational Interviewing to Improve Health-Related Behaviors: Scoping Review
Source: J Med Internet Res. 2023 Dec 8;25:e52097. doi: 10.2196/52097 (PMC10746972; doi:10.2196/52097)
Supplement: Multimedia Appendix 1 [file jmir_v25i1e52097_app1.docx]

**Multimedia Appendix 1.** Keywords used to find studies.

| **Database** | **Keywords** | **Hits** | % (n=404) |
| --- | --- | --- | --- |
| PubMed: <https://pubmed.ncbi.nlm.nih.gov/advanced/> | ( ( "Embodied Conversational Agent*" ) OR ( "Virtual Agent*" ) OR ( "Digital Health Agent*" ) OR ( "Virtual Assistant*" ) OR ( "Conversational Agent*" ) )  AND ( ( "Brief Motivational Interview*" ) OR ( "Motivational Interview*" )  OR ( "Brief Motivational Intervention*" ) OR ( "Motivational Intervention*" ) ) | 11 | 2.7 |
| Scopus <https://www.scopus.com/search/form.uri?display=advanced> | ( ( "Embodied Conversational Agent*" ) OR ( "Virtual Agent*" ) OR ( "Digital Health Agent*" ) OR ( "Virtual Assistant*" ) OR ( "Conversational Agent*" ) )  AND ( ( "Brief Motivational Interview*" ) OR ( "Motivational Interview*" )  OR ( "Brief Motivational Intervention*" ) OR ( "Motivational Intervention*" ) ) | 314 | 77.7 |
| IEEE <https://ieeexplore.ieee.org/search/advanced/command> | ( ( "Embodied Conversational Agent*" ) OR ( "Virtual Agent*" ) OR ( "Digital Health Agent*" ) OR ( "Virtual Assistant*" ) OR ( "Conversational Agent*" ) ) AND ( ( "Brief Motivational Interview*" ) OR ( "Motivational Interview*" ) OR ( "Brief Motivational Intervention*" ) OR ( "Motivational Intervention*" ) ) | 4 | 1.0 |
| ACM Digital <https://dl.acm.org/search/advanced> | ( ( "Embodied Conversational Agents" ) OR ( "Virtual Agents" ) OR ( "Digital Health Agents" ) OR ( "Virtual Assistants" ) OR ( "Conversational Agents" )  OR ( "Embodied Conversational Agent" ) OR ( "Virtual Agent" ) OR ( "Digital Health Agent" ) OR ( "Virtual Assistant" ) OR ( "Conversational Agent" ) )  AND ( ( "Brief Motivational Interviewing" ) OR ( "Motivational Interviewing" )  OR ( "Brief Motivational Interview" ) OR ( "Motivational Interview" )  OR ( "Brief Motivational Interviews" ) OR ( "Motivational Interviews" )  OR ( "Brief Motivational Intervention" ) OR ( "Motivational Intervention" )  OR ( "Brief Motivational Interventions" ) OR ( "Motivational Interventions" ) ) | 64 | 15.9 |
| PsyInfo <https://psycnet.apa.org/search/advanced> | ( ( "Embodied Conversational Agent*" ) OR ( "Virtual Agent*" ) OR ( "Digital Health Agent*" ) OR ( "Virtual Assistant*" ) OR ( "Conversational Agent*" ) ) AND ( ( "Brief Motivational Interview*" ) OR ( "Motivational Interview*" ) OR ( "Brief Motivational Intervention*" ) OR ( "Motivational Intervention*" ) ) | 0 | 0 |
| Other sources | Reference checking | 11 | 2.7 |
|  | Total: | 404 | 100 |
