# Supplementary material for: Embodied Conversational Agents Providing Motivational Interviewing to Improve Health-Related Behaviors: Scoping Review
Source: J Med Internet Res. 2023 Dec 8;25:e52097. doi: 10.2196/52097 (PMC10746972; doi:10.2196/52097)
Supplement: Multimedia Appendix 2 [file jmir_v25i1e52097_app2.docx]

**Multimedia Appendix 2.** Description of the data extraction fields.

| **Data extraction field** | **Description** |
| --- | --- |
| **Title** | The title of the article |
| **Author(s)** | The name of the authors in the article |
| **Year of publication** | The year of the publication of each article |
| **Country** | Country in which article published was noted |
| **Publication type** | Show whether studies are research articles of conference proceedings. |
| **Health problem addressed** | The health problem addressed through the ECA and MI |
| **ECA name** | The name assigned to the ECA in the article |
| **ECA appearance (gender)** | Represent the gender of the ECA.  Three categories:  1: Female  2: Male  3: Both (female and male) |
| **ECA appearance (ethnicity)** | Represent the appearance of the ECA.  Two categories:  1: No match the ethnicity of the users  2: Match the ethnicity of the users |
| **ECA dialogue mechanism** | Represent the dialogue mechanism implemented for conduct the MI intervention through the ECA.  Two categories:  1: Rules-driven  2: predefined or sequential |
| **ECA emotional model** | Represent how the emotional model was implemented  There are four categories:  1: Only shows emotions  2: Recognize user's emotion and show emotions  3: Not mentioned  4: Not implemented (A wizard of Oz study) |
| **ECA device implementation** | Represent which type of device was the ECA implemented for.  Two categories:  1: PC and Web-based PC  2: Tablet or Smartphone |
| **ECA implementation level** | ECAs were implemented at three levels:  1: Wizard-of-Oz  2: Prototype  3: Full System |
| **MI type** | Represent the type of MI implemented through the ECA.  Two categories:  1: MI  2: Brief MI |
| **MI implementation level** | The MI where implement at two levels:  1: As the core  2: As a component |
| **MI principles** | The MI principles implemented on the ECA.  There are four basic MI principles:  1: Empathy  2: Discrepancy  3: Roll with the resistance  4: Self-efficacy |
| **MI processes** | The MI processes implemented on the ECA.  There are four basic MI process:  1: Engaging  2: Focusing  3: Evoking  4: Planning |
| **MI techniques** | The MI techniques implemented on the ECA.  There are four basic MI techniques:  1: Open questions  2: Affirmations  3: Reflections  4: Summaries |
| **Level of the evaluation protocol** | Show the level of the evaluation protocol  Four categories:  1: Preliminary user study  2: Pilot study  3: Quasi-experimental study  4: Randomized Controlled Trials (RCT) |
| **Number of participants** | Represent the number of participants on the conducted study |
| **Participants profile** | Represent the profile of the participants on the conducted study |
| **Evaluation measures** | Show the evaluation measures reported in the article.  Five categories:  1: Feasibility  2: Acceptability / usability / user experience  3: Change on knowledge  4: Change on attitude, belief, or motivation  5: Change in behavior |
| **Main reported results** | The main results reported in the article |
