# Supplementary material for: Embodied Conversational Agents Providing Motivational Interviewing to Improve Health-Related Behaviors: Scoping Review
Source: J Med Internet Res. 2023 Dec 8;25:e52097. doi: 10.2196/52097 (PMC10746972; doi:10.2196/52097)
Supplement: Multimedia Appendix 3 [file jmir_v25i1e52097_app3.docx]

**Multimedia Appendix 3.** Details of the included studies.

| **Author** | **Year** | **Country** | **Publication type** | **Health problem addressed** | **ECA name** |
| --- | --- | --- | --- | --- | --- |
| Lisetti et al [41] | 2012 | United States | Conference Paper | Support reducing alcohol use | No name provided |
| Lisetti et al [36] | 2013 | United States | Journal Article | Support reducing alcohol use | ODVIC |
| Friederichs et al [28] | 2014 | The Netherlands | Journal Article | Promote physical activity | No name provided |
| Jack et al [26] | 2015 | United States | Journal Article | Promote women's preconception health | Gabby |
| Schouten et al [38] | 2018 | The Netherlands | Journal Article | Support cognitive learning | VESSEL |
| Olafsson et al [42] | 2019 | United States | Conference Paper | Promotion of fruit and vegetable consumption and physical activity | Emily and Katherine |
| Tielman et al [37] | 2019 | United States | Journal Article | Support reducing alcohol use | No name provided |
| Jack et al [27] | 2020 | United States | Journal Article | Promote women's preconception health | Gabby |
| Olafsson et al [43] | 2020 | United States | Conference Paper | Promotion of fruit and vegetable consumption and physical activity | Emily and Katherine |
| Olafsson et al [44] | 2020 | United States | Conference Paper | Counseling individuals in medication-assisted treatment for opioid use disorder. | No name provided |
| Boustani et al [39] | 2021 | United States | Journal Article | support reducing alcohol use | eEVA (framework) |
| Hocking and Maeder [45] | 2021 | Australia | Conference Paper | Support brain Injury rehabilitation decision-making processes | RehabChat |
| Rubin et al [29] | 2022 | United States | Journal Article | Support reducing alcohol use | No name provided |
| Schouten et al [40] | 2022 | The Netherlands | Journal Article | Support cognitive learning | VESSEL |
