# Supplementary material for: Embodied Conversational Agents Providing Motivational Interviewing to Improve Health-Related Behaviors: Scoping Review
Source: J Med Internet Res. 2023 Dec 8;25:e52097. doi: 10.2196/52097 (PMC10746972; doi:10.2196/52097)
Supplement: Multimedia Appendix 4 [file jmir_v25i1e52097_app4.docx]

**Multimedia Appendix 4.** Extended details of the evaluation protocol and main reported results.

| **Author** | **Evaluation protocol** | **Evaluation measures** | **Main reported results** |
| --- | --- | --- | --- |
| Lisetti et al [41] | A survey study (post- measurements), with 11 participants | - Acceptability / usability / user experience | The results suggest that employing ECAs for health-promoting interventions may result in higher user engagement, lower attrition rates compared to text-only computer-based health systems, and an overall increase in the disclosure of sensitive issues due to enhanced confidentiality. |
| Lisetti et al [36] | A comparative investigation was conducted involving three system versions: text-only, a non-empathic, and an empathic ECA (post-measurements), encompassing 81 users. Among these users, 26 were allocated to the empathic version, 25 to the non-empathic ECA, and 30 to the text-only version. | - Acceptability / usability / user experience | Results indicate that the widespread acceptance of the system by users, spanning different dimensions related to the impact of empathic communication from ECA, suggests that this method of delivering behavior change intervention would strongly affect users' motivation to persistently engage with computer-based interventions aimed at fostering the adoption and maintenance of healthy lifestyles over the long term. |
| Friederichs et al [28] | Conducted as a three-arm RCT with pre- and post-measurements, the study involved 958 individuals (578 females) who completed the questionnaire at baseline, along with the first follow-up after the intervention. The second follow-up measurements, taken one month later, were completed by 500 participants aged between 18 and 70 years old. | - Acceptability / usability / user experience - Change in behavior | Both interventions resulted in a noteworthy rise in self-reported physical activity within a month, when contrasted with the control condition. These findings imply that MI-based web interventions for physical activity hold potential for effectively instigating behavior change. However, no discernible differences emerged in terms of the impact on physical activity levels between the AVATAR and TEXT interventions. Likewise, the outcomes of evaluation did not reveal any significant variances among the two interventions. This absence of differentiation might be attributed to the avatar's inability to respond with gestures to the user's state and inputs. |
| Jack et al [26] | RCT (pre- and post- measurements), with 100 women (aged 18 to 34 years) | - Acceptability / usability / user experience - Change in behavior | The ECA possesses the ability to detect preconception risks and implement measures to mitigate them. Engaging with the ECA was notably linked to a larger proportion and a higher average count of preconception health risks being resolved within six months, in comparison with women who did not interact with the ECA. Participants belonging to the ECA group also conveyed that conversing with Gabby was comfortable, with the majority expressing their utilization of, or intentions to use, information provided by the ECA for enhancing their health. |
| Schouten et al [38] | Conducted as a pilot study involving 34 participants (19 to 64 years old), 20 men and 14 women. The experiment employed mixed-method repeated-measures, incorporating both within and between-subjects’ measurements. Pre- and post-assessments were carried out as part of the study design. | - Acceptability / usability / user experience - Change in knowledge - Change in attitude, belief, or motivation | The findings suggest that both prototypes operated according to expectations, with learners engaging with the coach and successfully completing all exercises. Nevertheless, minimal noteworthy distinctions were observed between the two prototypes, implying that the intended affective and social support might not have achieved the desired effectiveness. Furthermore, the results highlight significant disparities between two subsets of low-literate participants and among male and female participants. This emphasizes the significance of employing tailored support strategies for this specific demographic group. |
| Olafsson et al [42] | A Quasi-experimental study (pre- and post- measurements), with 39 participants (individuals at least 21 years old). A one-factor counterbalanced within-subjects experiment. | - Acceptability / usability / user experience - Change in attitude, belief, or motivation | Both interventions successfully induced changes in motivation, confidence, and self-efficacy related to modifying physical activity and fruit and vegetable consumption behaviors. Strong satisfaction with both ECAs and interventions was evident through both quantitative and qualitative self-reports. The ECAs demonstrate proficient implementation of motivational interviewing elements, assisting individuals in enhancing their motivation and confidence to initiate action. |
| Tielman et al [37] | Two pilot feasibility and validation studies (post-test measurement). Pilot 1 (post-test measurements), with 20 patients, and Pilot 2 (pre- and post-test measurements), with 30 participants. Participants are all males (aged 27 to 82 years). | - Feasibility - Acceptability / usability / user experience | Relational agents serve as an effective medium for substance use screening in primary care and are well accepted as an intervention modality. Specifically, veterans demonstrate comfort with the technology and a willingness to disclose sensitive information about alcohol use to the ECA. |
| Jack et al [27] | RCT (pre- and two recollections of data during the use of the ECA at 6 and 12 months), with 528 women (aged 18 to 34 years) | - Acceptability / usability / user experience - Change in behavior | Over the course of 6 months, employing the Gabby online system led to an increased proportion of reported preconception care risks progressing to the action or maintenance stages of the transtheoretical model, when compared to the control group. This effect persisted at the 12-month mark. Following the initial 6-month period, the majority of participants found Gabby's usage to be straightforward, expressing a strong sense of trust in the system, and confirming that Gabby effectively addressed their inquiries. Moreover, most participants reported having already implemented health changes based on Gabby's recommendations, while a few outlined their intentions to make health-related adjustments in the future. These findings remained consistent during the 12-month follow-up assessment. |
| Olafsson et al [43] | A quasi-experimental study encompassing pre- and post-measurements, the research involved 15 participants, all aged at least 21 years. The study adopted a two-treatment counterbalanced within-subjects experimental design. | - Acceptability / usability / user experience - Change in attitude, belief, or motivation | The findings suggest that an ECA incorporating affiliative and contextually fitting humor within a concise motivational interview can effectively boost individuals' motivation for both exercise and healthy eating. Additionally, humor emerges as a valuable tactic for fostering active involvement with an ECA. |
| Olafsson et al [44] | Feasibility Pilot Study (post- measurement), with 23 participants (aged 23 to 67 years). 22% were female. | - Acceptability / usability / user experience | The findings indicate that patients undergoing medication-assisted treatment generally exhibited favorable responses when engaging with a virtual agent to discuss matters concerning opioid use disorder therapy. They conveyed a notable level of trust in the ECA and expressed interest in future collaboration. On the whole, patients reported high level of satisfaction with the experience. Nevertheless, measurements evaluating the perceived closeness of the relationship with the agent yielded lower scores. The interviews indicated that initiating any form of relationship with the agent after just one session felt premature. |
| Boustani et al [39] | Descriptive Study (post- measurement), with 51 participants: 32 male and 19 female (aged 21 to 55 years) | - Feasibility - Acceptability / usability / user experience | Descriptive statistics revealed participants' highly positive encounters with the ECA, encompassing aspects like active involvement with the technology, perceived usefulness, acceptance, and intention to use it in subsequent instances. Elucidating qualitative quotes provided additional depth to the potential extent and influence of ECAs within the realm of behavioral healthcare. |
| Hocking and Maeder [45] | Feasibility study with alpha and beta testing phases (post- measurements): alpha testing with 3 participants, and beta testing with 11 participants. | - Feasibility - Acceptability / usability / user experience | The preliminary assessment of the ECA "RehabChat" involved evaluating its clinical importance and potential usability, while also pinpointing areas necessitating additional refinement. This process has substantiated the suitability of conversation and dialogue structuring, as well as the effectiveness of the two style variations. The outcomes from these testing phases serve as the groundwork for the planned enhancement and expansion of the ECA, which will be accomplished through collaborative co-design workshops and a subsequent pilot trial. |
| Rubin et al [29] | RCT (pre- and post-test measurements), with 178 veterans, 2 groups: TAU (treatment as usual) and Relational Agent + TAU. TAU: 79 male and 10 female; RA+TAU: 79 male and 10 female. | - Change in behavior | TheECA adeptly provided a concise intervention and significantly augmented patient referrals to specialized care, efficiently assisting individuals with less severe drinking issues without adding to the workload of primary care. Furthermore, the incorporation of ECA interventions has the potential to promote a more uniform implementation of SBIRT procedures. |
| Schouten et al [40] | Conducted as a preliminary study involving pre- and post-measurements, the research comprised 12 participants whose ages ranged from 30 to 63 years, with an equal split of 6 men and 6 women. The study adopted a mixed-method repeated-measures design that integrated within-subject analysis. The experiment encompassed two distinct conditions: one involving the ECA and the other without it. | - Acceptability / usability / user experience - Change in knowledge - Change in attitude, belief, or motivation | The findings suggest that the ECA yields noteworthy advantages for individuals with low literacy levels (utilizing VESSEL). The ECA profoundly impacts the subjective learning journey, as participants express elevated levels of positive emotions, heightened engagement with the user-system interaction, and enhanced self-efficacy concerning online banking. Furthermore, the outcomes reveal that low-literate users readily embraced the ECA as a valuable and dependable source of assistance. |
